# Recommendations to Optimize the Use of Volumetric MRI in Huntington's Disease Clinical Trials

**DOI:** 10.3389/fneur.2021.712565

**Published:** 2021-10-22

**Authors:** Kirsi M. Kinnunen, Ariana P. Mullin, Dorian Pustina, Emily C. Turner, Jackson Burton, Mark F. Gordon, Rachael I. Scahill, Emily C. Gantman, Simon Noble, Klaus Romero, Nellie Georgiou-Karistianis, Adam J. Schwarz

**Affiliations:** ^1^IXICO, London, United Kingdom; ^2^Critical Path Institute, Tucson, AZ, United States; ^3^Wave Life Sciences, Ltd., Cambridge, MA, United States; ^4^CHDI Management/CHDI Foundation, Princeton, NJ, United States; ^5^Teva Pharmaceuticals, West Chester, PA, United States; ^6^Huntington's Disease Research Centre, UCL Institute of Neurology, London, United Kingdom; ^7^School of Psychological Sciences and Turner Institute for Brain and Mental Health, Monash University, Melbourne, VIC, Australia; ^8^Takeda Pharmaceuticals, Ltd., Cambridge, MA, United States

**Keywords:** Huntington's disease, neurodegenerative, clinical trials, biomarkers, volumetric MRI, C-Path

## Abstract

Volumetric magnetic resonance imaging (vMRI) has been widely studied in Huntington's disease (HD) and is commonly used to assess treatment effects on brain atrophy in interventional trials. Global and regional trajectories of brain atrophy in HD, with early involvement of striatal regions, are becoming increasingly understood. However, there remains heterogeneity in the methods used and a lack of widely-accessible multisite, longitudinal, normative datasets in HD. Consensus for standardized practices for data acquisition, analysis, sharing, and reporting will strengthen the interpretation of vMRI results and facilitate their adoption as part of a pathobiological disease staging system. The Huntington's Disease Regulatory Science Consortium (HD-RSC) currently comprises 37 member organizations and is dedicated to building a regulatory science strategy to expedite the approval of HD therapeutics. Here, we propose four recommendations to address vMRI standardization in HD research: (1) a checklist of standardized practices for the use of vMRI in clinical research and for reporting results; (2) targeted research projects to evaluate advanced vMRI methodologies in HD; (3) the definition of standard MRI-based anatomical boundaries for key brain structures in HD, plus the creation of a standard reference dataset to benchmark vMRI data analysis methods; and (4) broad access to raw images and derived data from both observational studies and interventional trials, coded to protect participant identity. In concert, these recommendations will enable a better understanding of disease progression and increase confidence in the use of vMRI for drug development.

## Introduction

Like other neurodegenerative diseases, Huntington's disease (HD) is currently diagnosed by its clinical signs, despite its cardinal characteristic of being a fully penetrant monogenic disease (1). In recent years there has been a move toward specific pathobiological definitions of disorders, such as Alzheimer's disease (AD) (2), utilizing molecular biomarkers. Similar work is now taking place for HD (3, 4), and this shift will facilitate the translation of biological findings into therapeutic strategies. While molecular biomarkers of HD are under active development, quantitative imaging measures of brain structure from volumetric magnetic resonance imaging (vMRI) are already available. vMRI has been widely used in HD observational studies (5), demonstrating that loss of volume in HD-relevant brain regions (including caudate and putamen) is associated with clinical disease progression (6) and differences in brain volume are present in individuals with *HTT* CAG expansion prior to the presentation of clinical signs and symptoms as compared to age-matched controls (3, 4).

The potential value of longitudinal structural imaging in therapeutic trials is to provide biomarkers that can be used to assess pharmacodynamic effects of treatment on neurodegeneration (e.g., slowing of disease-related brain atrophy), in support of clinical outcome measures. vMRI is increasingly implemented in HD clinical trials, with sponsors that make use of it ranging from small biotech companies to large multinational pharmaceutical companies. The Huntington's Disease Regulatory Science Consortium (HD-RSC) is an initiative led by the Critical Path Institute and CHDI Foundation with 37 members from the biopharmaceutical industry, academia, and non-profit and patient-advocacy organizations that is generating drug development tools to define regulatory pathways and improve clinical trial efficiency in HD. Under the aegis of the HD-RSC, the imaging sub-team of the Biomarker Working Group, represented by the present authors, is tasked with assessing the clinical trial potential of leading candidate HD neuroimaging biomarkers. We recently reviewed the available evidence linking regional brain vMRI measurements to the biological and clinical characteristics of HD from the viewpoint of utility as biomarkers for clinical trials (7). That review concluded that a better understanding of the generalizability of reported longitudinal structural changes and their relationship to change in clinical outcomes was required, and that increased standardization of acquisition and analysis methods would be central to defining these aspects. Increased standardization will also enable more reliable quantitative descriptions of the disease process, which will further aid in the interpretation of clinical trial data to help establish universally interpretable volumetric values throughout HD clinical research.

Our aim here is to provide direction toward a more standardized use of vMRI in HD clinical research. Our core recommendations are: (1) a checklist of standardized practices for the use of vMRI and the reporting of results; (2) suggested investigations to resolve open questions on technical advances in MRI that could benefit the field; (3) a roadmap for the definition of standard MRI-based anatomical boundaries for key brain structures and the creation of a standard reference dataset to benchmark vMRI data analysis methods; and (4) a call for the routine sharing of raw images and derived image data from both interventional trials and observational studies, coded to protect participant identity. Adoption of these recommendations will help standardize data acquisition, analysis, and reporting, and thereby facilitate inter-study comparisons, meta-analyses, and *post hoc* data federation to greatly increase the value of vMRI in HD clinical research overall, including its utility in pathobiological disease staging (3).

## Recommendations for the Use of VMRI in HD Clinical Trials

We propose a checklist of recommendations that we encourage all HD research, especially clinical trials, to adopt (Table 1); these cover considerations of particular relevance to participants with HD, vMRI data acquisition, image analysis and publication of findings. While some of the recommendations are straightforward and familiar to imaging researchers, we have included key principles with the intent of helping all sponsors, including those with limited previous experience with imaging in an HD context.

**Table 1:** Recommendations for the use of vMRI in HD clinical trials.

**Considerations relevant to imaging participants with HD**
❑ Minimize motion-related artifacts and patient discomfort
° Minimize overall scanning time to limit movement artifacts that affect image quality. If long imaging sessions are required, include the option for a break between sequences to reduce discomfort.
° Take into account in booking the MRI machine and participant visit that the total scanning time can be considerably longer if the participant requires a break, or if sequences need to be repeated.
° Take time to ensure the patient is comfortable before the scanning session begins, using padding around the head and participant instruction to reduce movement.
° Where possible, rely on technologists with experience of scanning individuals with HD.
❑ Accommodate anxiety and cognitive impairment
° Explain the MRI processes to the trial participants sensitively and carefully. A rushed, fast-paced procedure is known to increase anxiety, and consequently in-scanner motion.
° Sedation may be considered and, if required, should be performed in line with the study's clinical protocol and the site's local MRI-sedation guidelines.
° If anti-anxiety or sedative medication is used, perform clinical assessments at a time when the medication will not interfere with the scan.
**Image acquisition**
❑ Use scanners with a 3T magnetic field where possible.
❑ Acquire the 3D T1W image in the sagittal plane, with approximately 1mm^3^ isotropic resolution, and adhere closely to widely-used acquisition protocols implemented in multi-site studies to ensure good gray/white matter contrast.
❑ Acquire two back-to-back 3D T1W scans to minimize missing data and additional visits for participants due to rescan requests.
❑ Include a research-grade diffusion MRI sequence capable, at minimum, to assess brain tissue microstructure through diffusion tensor-based analysis.
❑ Acquire any sequences that follow the T1W scan in order of their priority in the study, to reduce effects of positional changes or missing data.
❑ Perform on-site image QC to ensure full coverage of the brain, skull and cerebellum with field of view positioned to avoid wraparound artifacts, to detect artifacts due to motion, to verify the signal-to-noise ratio, and to check the uniformity of the signal intensity across the image. If necessary, repeat the scan immediately, to avoid a separate rescan visit.
❑ Ensure consistent positioning of the participant's head as near to the isocenter of the magnet as possible. This helps to reduce effects on image quality from geometric distortion caused by non-linear gradients (e.g., changes in head shape) that can necessitate a rescan. Also ensure consistent centering of the field-of-view (e.g., between the thalami).
❑ Perform the MRI exam prior to any lumbar puncture for cerebrospinal fluid sampling on the same day.
**Analysis and reporting of results**
❑ Quantify and report volumetric changes from multiple brain regions expected to be differentially affected in HD, including at minimum caudate, putamen, whole brain, lateral ventricles, cerebral white matter and hippocampus as a region expected to be minimally affected in HD.
❑ Report summary baseline values as (i) “raw” (unadjusted) measurements, (ii) fractions of total intracranial volume (TIV), and (iii) measurements adjusted (as appropriate) for age, TIV, and disease-related covariates such as CAG-repeat length and disease burden.
❑ Report longitudinal change for each treatment arm as percent of baseline value for each MRI outcome measure, as both (i) “raw” (unadjusted) and (ii) adjusted per the trial's statistical analysis plan, specifying which covariates were used in the statistical model.
❑ Compare placebo arm rate-of-change with similar cohorts from previous observational studies or interventional trials.
❑ Report treatment effects as percent slowing of decline relative to placebo or control arm and indicate whether the change favors treatment or placebo.
❑ Report statistical associations between regional volume measures and relevant clinical outcome measures both at baseline (cross-sectional) and longitudinally (change vs. change) in all arms.


MRI scanning in HD research participants after clinical motor diagnosis can be challenging due to involuntary motor symptoms that increase the risk of patient discomfort and motion artifacts in the images. Such artifacts can result in poor definition of brain structural boundaries, compromising the accuracy of region-of-interest segmentation and increasing the prevalence of image quality control failures; more severely affected patients [e.g., Unified Huntington's Disease Rating Scale (UHDRS) (8) and Total Functional Capacity (TFC) < 7] are rarely scanned for this reason and it should be acknowledged that this may introduce a bias since dropout may be more likely in patients with advanced disease. Statistical analysis can identify whether there are factors contributing to dropout [e.g., “Missing at Random” analysis (9)] and where necessary disease severity can be adjusted for in the design. In general, a consideration of the specific motor, behavioral, and cognitive problems HD individuals may exhibit and an emphasis on ensuring participant comfort during imaging visits has proved successful in maintaining image quality. Use of anxiolytic or sedative medication to minimize anxiety or motion during the scan should be recorded, and clinical outcome assessments should be either completed prior to medication or on a different study day. We outline the main recommendations (Table 1), including the strategies for addressing participant movement.

Centralized oversight of imaging procedures is a critical component of standardization in clinical research and will enable implementation of the recommendations (Table 1). There are several considerations, starting from the suitability of the imaging sites and scanners for the trial's MRI requirements, that may include additional sequences beyond the 3D T1-weighted (T1W) scan that is used for vMRI. In addition to the scanner manufacturer, make, model and magnetic field strength, differences in variables such as field homogeneities and image reconstruction routines can influence the data (10) and are important considerations when selecting sites for longitudinal studies. Image quality and its consistency across sites and over time determine the quality of analysis inputs and therefore the reliability of vMRI endpoint measurement. Higher magnetic field strength is usually associated with better quality of brain images, although some bias field artifacts are more visible. Given the current state of MRI scanner availability, we recommend the use of 3 Tesla (3T) scanners. The use of a 1.5T scanner may be acceptable for collecting vMRI as well as safety MRI data if no 3T scanner is available at an imaging center near the clinical site. However, if the study's MRI protocol also includes advanced sequences (e.g., diffusion MRI), this can compromise data quality. Using imaging protocols that have been designed with the goal of harmonization of image quality across scanner makes and models will also help to pool data in large-scale collaborations that aim to further our understanding of imaging biomarker trajectories in HD, given that the community will continue to increasingly commit to the sharing of imaging data from clinical research (see our recommendations in **Table 4**).

MRI sequence parameters should be harmonized as much as possible across participating sites, personnel trained on the imaging procedures, and the sites provided with clear documentation. Heterogeneity in the acquired data can affect the quality of the research both cross-sectionally and longitudinally; substantially different images acquired at different sites would increase data noise, which can become a critical confound if some sites recruit a specific participant group more often. From a longitudinal perspective, stable scanning parameters allow more precise measurement of volume change. Table 2 shows how T1W scan acquisition varied in the TRACK-HD/Track-On HD and PREDICT-HD non-interventional studies. Changes in key parameters affecting contrast and quality are marked in the table as different variants. Note that the number of T1W scan variants is higher both cross-sectionally and longitudinally in PREDICT-HD.

**Table 2:** A comparison of anatomical (T1W MRI) data homogeneity in two large multi-site observational studies in HD.

	TRACK-HD-BIDS^*^ (combined TRACK studies)	**PREDICT-HD-BIDS^≠^**
Number of sites:	4	32
Number of participants with T1W data:	448	1360
Number of MRI sessions with T1W data:	1,993	4,143
Average sessions per participant (N +/– SD):	4.4 +/– 2.0	3.0 +/– 2.4
Total T1W session-unique scans^i^:*(without T1W repetitions, but including different T1Ws on the same session)*	2,061	4,391
Total number of T1W variants in the study^ii^:!!!break *(lower = better)*	14	385
Number of common T1W variants^iii^:!!!break *(>2 occurrences in the study)*	8	82
Number of occurrences for common T1W variants^iv^:*(higher = better)*	2,054 (100%)	4,061 (92%)
Average occurrence rate for common variants^v^:!!!break *(higher = better)*	257 (13%)	50 (1%)
Average T1W voxel volume:*(lower = better)*	1.26 mm^3^	1.18 mm^3^
Standard deviation of T1W voxel volume^vi^:!!!break *(lower = better)*	0.10 mm^3^	0.31 mm^3^
Average T1W variants for each participant^vii^:!!!break *(lower = better)*	1.6 +/– 0.8	2 +/– 1.1
Participants with more than 1 T1W variant^viii^:!!!break *(lower = better)*	203 (45%)	802 (59%)
Participants with more than 2 T1W variants:!!!break *(lower = better)*	52 (12%)	412 (30%)

* *TRACK-HD-BIDS was created by combining data from TRACK-HD (four visits) and TrackOn-HD (three visits) studies. Nearly half of TRACK-HD participants were enrolled in TrackOn-HD; their sessions were concatenated in time. The merged dataset was converted into BIDS (Brain Imaging Data Structure) format, and the corresponding metadata and acquisition parameters were extracted from the json and imaging files*.

≠ *PREDICT-HD-BIDS contains data from the PREDICT-HD study, after converting the imaging data into the BIDS format. We extracted the metadata and acquisition parameters using the same routines for TRACK-HD-BIDS and PREDICT-HD-BIDS*.

*Only the T1W modality was investigated. Information relating to footnotes i-viii is provided in Appendix A*.

Consistent with natural history studies and trials in other neurodegenerative disorders, structural MRI scans in HD research have used 3D T1W sequences such as magnetization-prepared rapid acquisition with gradient echo (MP-RAGE) variants [e.g., TurboFLASH (Siemens), SPGR (GE) and T1-FFE (Philips)], which are widely accepted as standard for vMRI analyses. We recommend the use of these sequences for vMRI in HD, with the images acquired in the sagittal plane at 1 mm^3^ isotropic resolution. Other parameter settings should follow those established for a range of different scanners in widely referenced natural history studies in neurodegenerative disorders, maintaining good gray/white matter contrast. Generally, automatic segmentation pipelines can be more prone to failure due to lack of gray/white matter contrast rather than increased noise; thus, between sequences that yield less noise or more contrast, the latter is preferred, however segmentation methodology and region of interest should also be considered when optimizing a sequence. Slight variations in sequence parameters are sometimes necessary to accommodate different scanner types, software versions and coil/gradient hardware, and it is critical that these changes be carefully considered and centrally reviewed by the core imaging facility to ensure acceptable image quality. To reduce the amount of missing vMRI data and to avoid additional patient burden due to re-scan visits in a trial we specifically recommend acquiring two back-to-back 3D T1W scans in the same session. This will increase the likelihood that at least one T1W image is suitable for analysis, especially if care is taken to maintain consistent positioning across visits and to minimize head motion.

Since neurologic diseases carry particular challenges in MRI studies, sites in HD trials/studies should ideally have previous experience in scanning individuals with movement disorders. Within-participant changes in scanner, hardware, or software during a trial should be minimized; if a change is unavoidable, the core imaging facility should be notified in advance to enable site re-qualification and the effect of the scanner hardware and/or software change on the imaging endpoints to be evaluated (which could include scanning healthy volunteers both before and after the upgrade). Scanner changes should be tracked in the study database to assess effects on data quality and vMRI measurements, and appropriately handled in the statistical analysis.

A dedicated image quality control (QC) policy should be in place for each trial/study, including real-time QC at the site during the scan itself, followed by rapid centralized QC upon receipt by the core imaging facility. Specific procedures to remediate QC deviations in site performance must be in place, including potential re-scan procedures, which may differ between safety sequences and those used for primary and exploratory quantitative outcomes. Quality of the imaging data can be confirmed using phantom scans at study start and throughout (particularly after any equipment changes) to measure key parameters of field homogeneity, image contrast, and image distortion. Image processing should be conducted by a single central facility in a consistent and auditable (CFR21.11-compliant) manner while maximizing the tracking of data provenance. Image QC is traditionally performed through visual inspection, but automated approaches are increasingly developed and piloted to complement or fully replace current manual approaches. However, as image QC failure for a core sequence in a study can mean a re-scan request (and a repeated study visit) or result in data being excluded from analysis, the checks performed (automated and visual) and the pass/fail criteria need to be carefully considered during study setup which means most clinical trial applications still rely on visual quality control.

Consistent and detailed reporting of vMRI outcomes (irrespective of statistical significance) will be critical to both enable future meta-analyses and maximize the comparability of results from interventional trials. At minimum, vMRI outcomes should be reported in supplementary materials if no specific imaging publication is planned. Reporting results from several brain regions rather than just from the striatal regions provides a more complete picture of any treatment effect on the pattern of brain atrophy; for example, non-specific confounding effects on vMRI outcomes might result in an apparent slowing of atrophy that is independent of the baseline disease-related rates of atrophy, rather than a true disease modification effect. The regions listed are the minimum set we recommend reporting on (Table 1) but reporting results from more regions is encouraged; we include the hippocampus because it is generally only minimally affected in HD, but algorithms are well-established for its measurement due to its prominence in AD research. The rationale for reporting regions atrophied by different degrees in HD is to help disambiguate treatment effects that are consistent with a slowing of the disease process from non-specific changes that might reflect confounding effects such as fluid shifts or inflammation (11). It is also important to be explicit on the directionality of treatment-induced changes (favoring treatment or favoring placebo) as the natural directionality is different for outcomes such as ventricular volume (increase is worse) and those measuring parenchymal volumes or thicknesses (decrease is worse). Overall, our recommendations for reporting and analysis are motivated by the relatively sparse and inconsistent reported structural imaging results from interventional trials in neurodegenerative diseases generally.

We recommend that a product mode diffusion MRI sequence (at minimum a single-shell acquisition suitable for diffusion tensor modeling, with ≥32 diffusion-weighted (at *b* = 1,000 s/mm^2^) volumes and at least one reference volume with *b* = 0 acquired in a scan session of 8-10 min) is included in the research protocol to complement the vMRI and potentially indicate the possibility of potential confounding treatment effects on vMRI outcomes due to non-specific effects such as fluid shifts, edema, or inflammatory responses. A treatment-related deviation in the relationship between brain macrostructure (regional volumes or cortical thickness) and regional diffusivity metrics reflecting the microstructure may indicate a mechanism other than a modification of disease-related atrophy [7]. We note that data in this area are still emerging and the diffusion sequence is thus exploratory; as such, it can be included toward the end of the MRI protocol, consistent with our recommendations in Table 1.

For studies requiring both MRI scans and cerebrospinal fluid (CSF) collection, possible confounds to vMRI outcomes can be reduced by ensuring that the lumbar puncture is not performed immediately prior to the MRI scanning session. Clear evidence regarding the effect of lumbar puncture on vMRI outcomes is lacking, but the potential effects of CSF drainage on vMRI (and participant comfort due to potential post-lumbar puncture headaches) should be considered; when scheduled for the same protocol visit, we recommend that the lumbar puncture is performed after the MRI scan. If CSF collection must be performed prior to the MRI scan for scheduling purposes, then the MRI scan should be performed at least 24 h after the lumbar puncture; this timing should be kept consistent for all participants throughout the study.

We have attempted to strike a balance between these recommendations being specific and readily actionable, while maintaining scientific and operational flexibility for individual trials/studies and sites. Recommendations can be periodically reviewed as scientific and technical advances warrant, and with a frequency appropriate to preserving inter-trial comparability.

## Recommended Further Work Toward Improved Image Acquisition

Standardizing and optimizing structural MRI acquisition protocols for multi-center studies has not been a focus of any single initiative in HD. We recommend that a dedicated study be undertaken to generate the empirical evidence required to determine the acquisition parameters across a range of scanners (manufacturers/models) that will optimize the quality of HD-relevant vMRI biomarkers. This investigation should take advantage of recent technical advances while ensuring practicability for clinical trials, in which many imaging sites are not high-end MRI academic research centers. Questions to be considered as a priority include the use of accelerated sequences, the best way to mitigate head motion, and the merits of increasing the spatial resolution relative to the current standard of 1 mm^3^ isotropic.

Accelerated 3D T1W sequences can almost halve scanning time and are now widely used in AD clinical trials following a head-to-head comparison with non-accelerated sequences within the Alzheimer's Disease Neuroimaging Initiative (ADNI) consortium; these investigations found no significant differences in whole-brain, ventricular and hippocampal atrophy rates measured from accelerated vs. non-accelerated scans (12, 13), and little effect on measured whole-brain atrophy values after switching from non-accelerated scans at baseline to accelerated scans at follow-up (14). However, vMRI outcomes of interest in HD have not been similarly compared so we recommend that when accelerated sequences are used they are acquired for both back-to-back scans to ensure consistency when either scan is used for analysis.

If found reliable and feasible to deploy in multi-site trials, technologies such as in-scanner motion detection/correction hardware or motion-corrected 3D T1W sequences (15) could offer substantial advantage. These tools are not yet available at most clinical imaging sites and the effect of real-time motion correction on volumetric measurements has not been established.

Increased spatial resolution would allow finer anatomical detail to be resolved, but the trade-off is a reduction in signal-to-noise ratio or an increase in scanning time. Understanding the improvement in volumetric accuracy derived from high-resolution images will be the first step in deciding whether this trade-off is warranted.

## Universally Applicable Anatomical Boundaries and A Reference Dataset to Benchmark Analysis Methods

Various software tools and image analysis algorithms have been used for regional brain segmentation and volumetric change measurement, and these techniques will continue to evolve. Quantitative volumetric assessment of key brain structures such as caudate and putamen has been a focus of HD clinical research, but there is no standard definition of their structural boundaries on MRI scans and different algorithms are trained with respect to different segmentation protocols. This likely contributes to the variance in reported volumes and atrophy rates, limits comparison between studies, and creates difficulty for the inclusion of these measures in biologically based disease models.

We propose that the HD research community establish consensus definitions of the anatomical boundaries of each structure of interest from MRI scans (Table 3), similar to that employed in the European Alzheimer's Disease Consortium–ADNI project to standardize hippocampal segmentation (16–19). Thereafter, structures of interest would be manually delineated by trained tracers on images from a standard reference dataset that will be made publicly available, consistent with open science principles. The manually segmented images in the standard reference dataset would serve as a neutral, non-proprietary gold standard against which any automated algorithm can then be benchmarked for segmentation accuracy. The benchmarking process should include appropriate statistical methods to quantify spatial overlap (e.g., Dice coefficient, Hausdorff distance, or average contour displacement) and the bias and variability (e.g., Bland-Altman analysis) of the prospective algorithm and the reference segmentations (20).

**Table 3:** Roadmap toward the definition of standard MRI-based anatomical boundaries.

**Cross-sectional use**
❑Establish expert consensus definition of MRI structural boundaries for key brain structures, with a focus on the caudate and putamen initially.
❑Select appropriate reference set of 3D T1W images.
❑Manually trace boundaries of selected structures in reference image set using consensus protocol.
❑Make reference images, structural boundaries, calculated volumes, and relevant documentation publicly available.
**Longitudinal use**
❑Select or develop, and make publicly available, an automated algorithm optimized for measurement of longitudinal change.
❑Select appropriate reference set of baseline and follow-up 3D T1W images (images could overlap with cross-sectional set).
❑Apply reference algorithm to longitudinal reference images.
❑Make reference images, measurements of volumetric change, and relevant documentation publicly available.
**Performance benchmarks for prospective automated algorithms**
❑Quantify spatial overlap between prospective algorithm segmentations and reference masks.
❑Determine linear regression relationship between reference values (segmented volumes or change measures) and those calculated by prospective algorithm.
❑Assess bias and variability of prospective algorithm with respect to reference values.
❑Evaluate prospective algorithm consistency using repeat analyses on same data.

For purposes of cross-sectional analyses or staging individuals based on their degree of brain atrophy, volumetric measurements of brain structures should be corrected by a measurement of intracranial volume (ICV) as a measure of head size (which is independent of the HD process). The estimate of ICV is usually obtained from the same 3D T1W image, but there is no established standard method for this. We therefore recommend that a reference algorithm for the measurement of ICV is established; this could be an existing algorithm or a novel one developed from the standard reference dataset.

To mimic clinical trial conditions, the standard reference images should originate from different sites and scanners and have the resolution and contrast required to allow a thorough technical evaluation of vMRI acquisition on measurement performance (scanner manufacturer and model, including differences in bore size, receiver coil type, accelerated acquisition methods, and field strength). Standard reference images should also span the spectrum of disease progression to account for different target populations in clinical trials. Due to their importance in HD and presumed tractability to achieve a consensus definition, we propose caudate and putamen as initial regions of interest; the process can then be extended to other brain regions. Access to the standard reference dataset must be straightforward, and relevant demographic and clinical information (e.g., sex, age, CAG-repeat length, scores from clinical rating scales such as UHDRS-TFC for staging and full UHDRS) should also be included. Under ethics board-approved protocols, all participants included in the standard reference dataset must have given informed consent for public access to the minimum required set of clinical and imaging data and their use in secondary analyses.

An algorithm's ability to reliably assess volume changes over time is crucial to its application as a treatment response biomarker in clinical trials. While suitable as a reference standard for cross-sectional analyses, manual tracing of structures (as proposed above) will be susceptible to additional intra-rater (and potentially also inter-rater) variability when applied to longitudinal measurements. We therefore propose that a reference automated vMRI algorithm, optimized for volume change measurement, is carefully selected and tested (or developed and validated) for the specific purpose of benchmarking other algorithms for longitudinal HD research. The reference algorithm would be applied to a set of baseline and follow-up images and included in the openly available standard reference dataset. This standard reference algorithm should be available for use by any trial sponsor or delegate (e.g., an imaging CRO), and can be reviewed by regulatory agencies so that sponsors can use it with confidence to directly derive imaging analysis metrics or to benchmark the performance of a proprietary algorithm.

Rather than explicitly endorsing specific algorithms, we are recommending a *process* whereby any algorithm can be tested against technical performance metrics. As part of this, specific numeric criteria for acceptable performance must be established alongside the preparation of the standard reference dataset.

## Broad Sharing of Imaging Data From Interventional Trials and Observational Studies

We propose that interventional clinical trials and observational studies be planned and consented with open science practices in mind (Table 4) (21). For example, the publicly available ADNI data have been extremely useful in advancing AD research and planning clinical trials (22–24), and the HD research community is now providing access to harmonized observational datasets from studies such as Enroll-HD, IMAGE-HD, PREDICT-HD, and TRACK-HD. Using comparable data from several datasets is critical to assess the variability and generalizability of findings across studies and more robustly inform clinical trial design. Previously acquired data can potentially be used as historical controls to combine with prospectively acquired interventional data and could enhance statistical power, especially in highly invasive trials with small sample sizes.

**Table 4:** Broad sharing of imaging data from interventional trials and observational studies.

❑Commit to open science data sharing principles at point of study conception and at organizational level.
❑Ensure study informed consent explicitly covers GDPR-mandated language for data sharing and secondary analysis to enable broad sharing of raw images, derived image data, and clinical/demographic variables.
❑Make images and other data compliant with HIPAA and GDPR.
❑Provide data in standard format (e.g., BIDS, CDISC).
❑For interventional trials, at minimum make placebo arm data available.

To use datasets for such purposes it is essential that study consent covers national and international privacy laws, including the General Data Protection Regulation (GDPR) requirement for explicit consent to share data, and includes language to enable broad sharing of both raw measurements and images, explicitly allowing secondary use of the data. Participant-level data, including images, should be made available through a neutral party; this will require participant identity protection that complies with GDPR as well as the Health Insurance Portability and Accountability Act (HIPAA). C-Path has previously published recommendations for effective sharing of participant-level data to advance medical product development while maintaining data security and patient privacy (21). Image data should be provided in a standard neuroimaging format that includes all the acquisition parameters necessary for image processing.

Data coding to protect participant identity is a critical and necessary step to enable seamless collation of data from multiple sources. In addition to image header fields that may contain identifying information, there is an increased awareness that imaging data can be used to render 3D photographic facial features with high accuracy (25). Consequently, we recommend that facial features are removed or blurred prior to making imaging data publicly available, using a method that does not unduly affect the quantification of the vMRI outcome measures.

It is important to emphasize the value of data from interventional clinical trials, which typically have more frequent imaging assessments than observational studies, represent true real-world examples from drug development, and may include treatment effects on disease-related biological changes. Sponsors should share both derived and raw image data from interventional trials once their analyses and obligations (e.g., intervention approved/terminated, results reported) are complete, and they should initiate this process as soon as the study database is locked. The process of data integration, storage, and management could be administered by a neutral third party; C-Path is already federating such data from interventional trials and has extensive experience in these quantitative analyses. Imaging data could be distributed as NIfTI and json files in a standardized format [e.g., Brain Imaging Data Structure (BIDS)] that facilitates data sharing and provides better control of participant identity. Phenotypic scores should also adhere to standardized formats [e.g., those specified by the Clinical Data Interchange Standards Consortium (CDISC) (26, 27)].

## Conclusions

The HD drug development community must address several gaps in the standardization and optimization of vMRI to obtain maximum value from measures of brain structure as quantitative biomarkers. While a degree of *de facto* standardization is in place for data acquisition, clear guidelines to manage the whole process have been lacking. Differences in segmentation techniques point to the need for standardized methods for both acquisition and analysis, which in turn will facilitate linking these quantitative metrics to established clinical and functional measures and the definition of biologically based disease models that include brain imaging markers. The four sets of recommendations proposed here directly address standardization of methodology (without privileging any one manufacturer or algorithm), which will help to maximize biological insight.

## Huntington's Disease Regulatory Science Consortium Coordinating Committee

Varun Aggarwal, Shazia Ali, Irina Antonijevic, Astri Arnesen, Nazem Atassi, Brian Beers, Beth Belluscio, Limor Ben Har, Angele Benard, Caroline Benn, Brian Bettencourt, Anu Bhattacharyya, Robi Blumenstein, Beth Borowsky, Bret Bostwick, Jackson Burton, Angelika Caputo, David Cooper, Brad Elmer, Rebecca Evans, Andrew Feigen, Terrence Fisher, Rebecca Fuller, Emily Gantman, Danielle Gartner, Michal Geva, Nellie Georgiou-Karistianis, Sandra Gonzalez, Adam Good, Mark Gordon, Jaya Goyal, Michael Hayden, Priyantha Herath, Steve Hersch, Jianying Hu, Elise Kayson, Eileen Koski, Bernhard Landwehrmeyer, Michelle Lax, Blair Leavitt, Dorothy Leong, Oren Levy, Enchi Liu, Jeff Long, Doug Macdonald, Jacqueline Major, Lahar Mehta, Tiago Mestre, Eric Miller, Christian Mueller, Catherine O'Riordan, Jennifer Panagoulias, Mike Panzara, Anne Pedata, Jennifer Petrillo-Billet, Dave Podskalny, Alisha Reader, Shelly Redman, Ralf Reilmann, Klaus Romero, Christopher Ross, Anne Rosser, Cristina Sampaio, Jan Samzelius, Scott Schobel, Adam Schwarz, Sudhir Sivakumaran, Jennie Socha, Glenn Stebbins, Julie Stout, Sarah Tabrizi, Emily Turner, Charles Venuto, Louise Vetter, Vissia Viglietta, Sarah Wahlstrom Helgren, Beth White, Ed Wild, George Yohrling, Maurice Zauderer.

The Huntington's Disease Regulatory Science Consortium is funded by CHDI Foundation, Inc., a nonprofit biomedical research organization exclusively dedicated to collaboratively developing therapeutics for Huntington's disease. The studies cited in this manuscript would not be possible without the vital contribution of the research participants and their families.

## Author Contributions

KMK, APM, NG-K, and AJS conceptualized the manuscript. KMK, APM, DP, ECT, JB, MFG, RIS, ECG, SN, KR, NG-K, and AJS drafted and revised the manuscript. All authors contributed to the article and approved the submitted version.

## Conflict of Interest

KMK is a full-time employee of IXICO. APM is a full-time employee of Wave Life Sciences, Ltd. DP, ECG, and SN are employed by CHDI Management to provide advisory services to CHDI Foundation. MFG is a full-time employee of Teva Pharmaceuticals. RIS provides consultancy for IXICO. AJS is a full-time employee and shareholder of Takeda Pharmaceuticals, Ltd. The remaining authors declare that the research was conducted in the absence of any commercial or financial relationships that could be construed as a potential conflict of interest.

## Publisher's Note

All claims expressed in this article are solely those of the authors and do not necessarily represent those of their affiliated organizations, or those of the publisher, the editors and the reviewers. Any product that may be evaluated in this article, or claim that may be made by its manufacturer, is not guaranteed or endorsed by the publisher.

## Abbreviations

3T: 3 Tesla
AD: Alzheimer's disease
ADNI: Alzheimer's Disease Neuroimaging Initiative
BIDS: Brain Imaging Data Structure
C-Path: Critical Path Institute
CDISC: Clinical Data Interchange Standards Consortium
CSF: Cerebrospinal fluid
dMRI: Diffusion Magnetic Resonance Imaging
EADC-ADNI: European Alzheimer's Disease Consortium—Alzheimer's Disease Neuroimaging Initiative
GDPR: General Data Protection Regulation
HD: Huntington's disease
HD-RSC: Huntington's Disease Regulatory Science Consortium
HIPAA: Health Insurance Portability and Accountability Act
ICV: Intracranial volume
MP-RAGE: Magnetization-prepared rapid acquisition with gradient echo
MRI: Magnetic resonance imaging
QC: Quality control
T1W: T1-weighted
TFC: Total Functional Capacity
UHDRS: Unified Huntington's Disease Rating Scale
vMRI: Volumetric magnetic resonance imaging.

## Appendix A

Footnotes for Table 2:

^i^Each session typically uses the same scanning parameters even when the T1W acquisition is repeated multiple times. We automatically eliminated the repeated sequences within each session but kept the T1W sequences if they differed within the session. Thus, we count “session-unique” scans.

^ii^A variant is defined when all the following parameters are the same: (1) voxel dimension, (2) field of view (FOV), (3) magnetic field strength (i.e., 1.5T and 3T), (4) scanner manufacturer, (5) scanner model, (6) repetition time (TR), (7) echo time (TE), and (8) flip angle. Any change in these parameters would constitute a new variant. Voxel and FOV dimensions are sorted incrementally to avoid the order of the values be mistaken as a different variant.

^iii^Common variants are identified by keeping only variants with more than two occurrences. Some T1W variants occur only 1-2 times due to protocol errors or specific site needs and may inflate the number of variants without substantially impacting the homogeneity of the data.

^iv^Data are more homogeneous if common variants cover the majority of session-unique scans.

^v^The average occurrence rate of common variants gives an indication of data harmonization in the study. Common variants should ideally be used in many MRI sessions on average. In TRACK-HD, each common variant accounts on average for 13% of all session-unique scans. In PREDICT-HD, each common variant accounts on average for 1% of all session-unique scans.

^vi^Even when there are many variants, having voxels of similar size might improve data homogeneity. Higher variance of voxel volumes indicates that variants produce images that are more different from one variant to another.

^vii^Using the same T1W variant at each participant visit is important for the correct interpretation of longitudinal volume changes. Ideally, each participant would undergo the same T1W variant at all longitudinal visits. When this value is higher than 1, it means that some participants have undergone multiple variants at their longitudinal visits.

^viii^Knowing how many participants have undergone more than one variant during longitudinal visits can help understand the extent of longitudinal de-harmonization. In the case of TRACK-HD, we combined two formally distinct studies and a variant change can be expected. In PREDICT-HD, participants should have followed the same variant, but there is more longitudinal heterogeneity than in TRACK-HD.

## References

[1] Huntington's Disease Collaborative Research Group. A novel gene containing a trinucleotide repeat that is expanded and unstable on Huntington's disease chromosomes. The Huntington's Disease Collaborative Research Group. Cell. (1993) 72:971–83. 10.1016/0092-8674(93)90585-E 8458085

[2] Jack CR Bennett DA Blennow K Carrillo MC Dunn B Haeberlein SB. NIA-AA research framework: toward a biological definition of Alzheimer's disease. Alzheimers Dement. (2018) 14:535–62. 10.1016/j.jalz.2018.02.018 29653606 PMC5958625

[3] Tabrizi SJ Schobel S Gantman EC Mansbach A Borowsky B Konstantinova P. Huntington's disease integrated staging system (HD-ISS): a novel evidence-based classification system for staging. medRxiv [Preprint]. (2021). Available online at: https://www.medrxiv.org/content/10.1101/2021.09.01.21262503v1

[4] Scahill RI Zeun P Osborne-Crowley K Johnson EB Gregory S Parker C. Biological and clinical characteristics of gene carriers far from predicted onset in the Huntington's disease Young Adult Study (HD-YAS): a cross-sectional analysis. Lancet Neurol. (2020) 19:502–12. 10.1016/S1474-4422(20)30143-5 32470422 PMC7254065

[5] Scahill RI Andre R Tabrizi SJ Aylward EH. Structural imaging in premanifest and manifest Huntington disease. Handb Clin Neurol. (2017) 144:247–61. 10.1016/B978-0-12-801893-4.00020-1 28947121

[6] Tabrizi SJ Langbehn DR Leavitt BR Roos RA Durr A Craufurd D. Biological and clinical manifestations of Huntington's disease in the longitudinal TRACK-HD study: cross-sectional analysis of baseline data. Lancet Neurol. (2009) 8:791–801. 10.1016/S1474-4422(09)70170-X 19646924 PMC3725974

[7] Kinnunen KM Schwarz AJ Turner EC Pustina D Gantman EC Gordon MF. Volumetric MRI-based biomarkers in Huntington's disease: an evidentiary review. Front Neurosci. (2021) 12:1552. 10.3389/fneur.2021.712555 34621236 PMC8490802

[8] Unified Huntington's Disease Rating Scale: reliability and consistency. Huntington Study Group. Mov Disord. (1996) 11:136–42. 10.1002/mds.870110204 8684382

[9] van Buuren S. Flexible Imputation of Missing Data. 2nd ed. Boca Raton: CRC Press, Taylor & Francis Group (2018).

[10] Van Horn JD Toga AW. Multisite neuroimaging trials. Curr Opin Neurol. (2009) 22:370–8. 10.1097/WCO.0b013e32832d92de 19506479 PMC2777976

[11] Schwarz AJ. The use, standardization, and interpretation of brain imaging data in clinical trials of neurodegenerative disorders. Neurotherapeutics. (2021) 18:686–708. 10.1007/s13311-021-01027-4 33846962 PMC8423963

[12] Hua X Ching CRK Mezher A Gutman BA Hibar DP Bhatt P Leow AD. MRI-based brain atrophy rates in ADNI phase 2: acceleration and enrichment considerations for clinical trials. Neurobiol Aging. (2016) 37:26–37. 10.1016/j.neurobiolaging.2015.09.018 26545631 PMC4827255

[13] Manning EN Leung KK Nicholas JM Malone IB Cardoso MJ Schott JM. Alzheimer's disease neuroimaging initiative. A comparison of accelerated and non-accelerated MRI scans for brain volume and boundary shift integral measures of volume change: evidence from the ADNI dataset. Neuroinformatics. (2017) 15:215–26. 10.1007/s12021-017-9326-0 28316055 PMC5443885

[14] Leung KK Malone IM Ourselin S Gunter JL Bernstein MA Thompson PM. Alzheimer's disease neuroimaging initiative. Effects of changing from non-accelerated to accelerated MRI for follow-up in brain atrophy measurement. Neuroimage. (2015) 107:46–53. 10.1016/j.neuroimage.2014.11.049 25481794 PMC4300278

[15] Tisdall MD Reuter M Qureshi A Buckner RL Fischl B van der Kouwe AJW. Prospective motion correction with volumetric navigators (vNavs) reduces the bias and variance in brain morphometry induced by subject motion. Neuroimage. (2016) 127:11–22. 10.1016/j.neuroimage.2015.11.054 26654788 PMC4754677

[16] Frisoni GB Jack CR Bocchetta M Bauer C Frederiksen KS Liu Y. The EADC-ADNI Harmonized Protocol for manual hippocampal segmentation on magnetic resonance: evidence of validity. Alzheimers Dement. (2015) 11:111–25. 10.1016/j.jalz.2014.05.1756 25267715 PMC4422168

[17] Duchesne S Valdivia F Robitaille N Mouiha A Valdivia FA Bocchetta M. Manual segmentation qualification platform for the EADC-ADNI harmonized protocol for hippocampal segmentation project. Alzheimers Dement. (2015) 11:161–74. 10.1016/j.jalz.2015.01.002 25617509

[18] Boccardi M Bocchetta M Apostolova LG Barnes J Bartzokis G Corbetta G. Delphi definition of the EADC-ADNI harmonized protocol for hippocampal segmentation on magnetic resonance. Alzheimers Dement. (2015) 11:126–38. 10.1016/j.jalz.2014.02.009 25130658 PMC4419736

[19] Jack CR Barkhof F Bernstein MA Cantillon M Cole PE Decarli C. Steps to standardization and validation of hippocampal volumetry as a biomarker in clinical trials and diagnostic criterion for Alzheimer's disease. Alzheimers Dement. (2011) 7:474–85.e4. 10.1016/j.jalz.2011.04.007 21784356 PMC3396131

[20] Raunig DL McShane LM Pennello G Gatsonis C Carson PL Voyvodic JT. Quantitative imaging biomarkers: a review of statistical methods for technical performance assessment. Stat Methods Med Res. (2015) 24:27–67. 10.1177/0962280214537344 24919831 PMC5574197

[21] Karpen SR White JK Mullin AP O'Doherty I Hudson LD Romero K. Effective data sharing as a conduit for advancing medical product development. Ther Innov Regul Sci. (2021) 55:591–600. 10.1007/s43441-020-00255-8 33398663 PMC7780909

[22] Weiner MW Veitch DP Aisen PS Beckett LA Cairns NJ Cedarbaum J. Impact of the Alzheimer's disease neuroimaging initiative, 2004 to 2014. Alzheimers Dement. (2015) 11:865–84. 10.1016/j.jalz.2015.04.005 26194320 PMC4659407

[23] Veitch DP Weiner MW Aisen PS Beckett LA Cairns NJ Green RC. Understanding disease progression and improving Alzheimer's disease clinical trials: recent highlights from the Alzheimer's Disease neuroimaging initiative. Alzheimers Dement. (2019) 15:106–52. 10.1016/j.jalz.2018.08.005 30321505

[24] Liu E Luthman J Cedarbaum JM Schmidt ME Cole PE Hendrix J. Perspective: the Alzheimer's disease neuroimaging initiative and the role and contributions of the private partner scientific board (PPSB). Alzheimers Dement. (2015) 11:840–9. 10.1016/j.jalz.2015.04.001 26194317

[25] Schwarz CG Kremers WK Therneau TM Sharp RR Gunter JL Vemuri P. Identification of anonymous MRI research participants with face-recognition software. N Engl J Med. (2019) 381:1684–6. 10.1056/NEJMc1908881 31644852 PMC7091256

[26] Mullin AP Corey D Turner EC Liwski R Olson D Burton J. Standardized data structures in rare diseases: CDISC user guides for duchenne muscular Dystrophy and Huntington's Disease. Clin Transl Sci. (2020) 14:214–21. 10.1111/cts.12845 32702147 PMC7877853

[27] Clinical Data Interchange Standards Consortium. Huntington's Disease Therapeutic Area User Guide v1.0. Available online at: https://www.cdisc.org/standards/therapeutic-areas/huntingtons-disease (accessed January 15, 2021).

